# Ketogenic diet for mitochondrial disease: potential role in treating the Multiple Symmetric Lipomatosis phenotype associated with the common MT-TK genetic mutation

**DOI:** 10.1186/s13023-021-02164-x

**Published:** 2022-01-10

**Authors:** Andre Mattman, Elizabeth Nadeau, Michelle M. Mezei, Mark Cresswell, Sida Zhao, Taryn Bosdet, Don D. Sin, Jordan A. Guenette, Isabelle Dupuis, Emily Allin, David C. Clarke

**Affiliations:** 1 Adult Metabolic Diseases Clinic, Vancouver General Hospital and Department of Pathology, Faculty of Medicine, Gordon & Leslie Diamond Health Care Centre, University of British Columbia, 4th Floor, 2775 Laurel Street, Vancouver, BC V5Z 1M9 Canada; 2 Vancouver, Canada; 3 Adult Metabolic Diseases Clinic, Vancouver General Hospital and Division of Neurology, Faculty of Medicine, University of British Columbia, 4th Floor, 2775 Laurel Street, Vancouver, BC V5Z 1M9 Canada; 4 Department of Radiology, St Paul’s Hospital, 1081 Burrard St, Vancouver, BC V6Z 1Y6 Canada; 5 Department of Biomedical Physiology & Kinesiology, Simon Fraser University, 8888 University Drive Burnaby, V5A 1S6, Burnaby, B.C. Canada; 6 Adult Metabolic Diseases Clinic, Vancouver General Hospital, 4th Floor, 2775 Laurel Street, Vancouver, BC V5Z 1M9 Canada; 7 Centre for Heart Lung Innovation, St Paul’s Hospital and, Division of Respiratory Medicine, Faculty of Medicine, University of British Columbia, 1081 Burrard St, Vancouver, BC V6Z 1Y6 Canada; 8 Centre for Heart Lung Innovation, St Paul’s Hospital and Department of Physical Therapy, Faculty of Medicine, University of British Columbia, 1081 Burrard St, Vancouver, BC V6Z 1Y6 Canada

Dear Editor,

We read with appreciation the succinct and informative systematic review by Zweers et al. [1] on the effect of the Ketogenic Diet and/or the Modified Atkins Diet on various mitochondrial disease associated phenotypes.

While this review covered studies pertaining to epilepsy, skeletal and heart muscle, tonus dysregulation, movement disorder, developmental delay/intellectual disability, food intake, weight gain/growth, and hair growth, we note that the multiple symmetric lipomatosis phenotype, frequently associated with the MT-TK genetic mutation, was not covered.

We would like to highlight that a version of the ketogenic diet was employed successfully, along with other simultaneous lifestyle interventions, in treating a disabling multiple symmetric lipomatosis phenotype. This successful intervention was reported in 2020 [2] and warrants further consideration by care providers whose patients are in a similar predicament, and similarly motivated.

Sincerely,
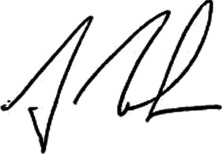


Andre Mattman, MD, FRCPC

Adult Metabolic Diseases Clinic

Vancouver, Canada.

## Data Availability

N/A.
